# Impact of a low‐carbohydrate diet on cardiometabolic parameters in individuals with and without diabetes: An 8‐year longitudinal study within the ELSA‐Brasil cohort

**DOI:** 10.1002/ncp.70099

**Published:** 2026-02-06

**Authors:** Leticia Batista de Azevedo, Haysla Xavier Martins, Oscar Geovanny Enriquez‐Martinez, Maria del Carmen Bisi Molina

**Affiliations:** ^1^ Postgraduate Program in Nutrition and Health Federal University of Espírito Santo Vitória Brazil; ^2^ Postgraduate Program in Public Health Federal University of Espírito Santo Vitória Brazil; ^3^ Postgraduate Program in Nutrition and Longevity Federal University of Alfenas Alfenas Brazil

**Keywords:** cardiovascular health, chronic diseases, diabete, diets, longitudinal study, public health

## Abstract

**Background:**

Low‐carbohydrate diets (LCDs) are widely adopted for metabolic management, but their long‐term cardiometabolic effects in diverse populations remain unclear. We aimed to evaluate the association between self‐selected moderate LCD adherence and cardiometabolic parameters in individuals with and without diabetes.

**Methods:**

This prospective cohort study included 9658 participants from the Brazilian Longitudinal Study of Adult Health (ELSA‐Brasil; baseline: 2008–2010; follow‐up: 2017–2019). LCD adherence (carbohydrate <45% of total energy) was assessed through validated food frequency questionnaires. Quantile regression models, adjusted for sociodemographic, lifestyle, and clinical factors, were used to analyze changes in body mass index (BMI), blood pressure, insulin, and homeostatic model assessment for insulin resistance (HOMA‐IR) and beta cell function (HOMA‐B).

**Results:**

Among individuals without diabetes, those who followed an LCD only at the follow‐up showed decreased insulin levels (β = −0.33 [95% CI, −0.6 to −0.1] μIU/ml), HOMA‐IR (−0.11 [−0.2 to −0.1] μIU/ml), and HOMA‐B (−4.95 [−7.2 to −2.1] μIU/ml) alongside modest BMI increases (0.16 [0.1–0.3]). Consistent LCD adherents (at both time points) showed a decrease in HOMA‐B (−3.21 [−6.4 to −0.1] μIU/ml) and an increase in BMI (0.29 [0.2–0.4]). In individuals with diabetes, LCD adherence led to reduced HOMA‐IR (−1.25 [−2.2 to −0.3] μIU/ml) and insulin levels (−3.61 [−6.0 to −1.2] μIU/ml).

**Conclusion:**

Moderate LCD adherence improved insulin sensitivity and reduced pancreatic demand in individuals without diabetes. Despite slight BMI increases, LCD may be a feasible dietary strategy for metabolic risk management in middle‐aged and older adults. Personalized nutrition approaches are recommended to optimize outcomes.

## Graphical abstract

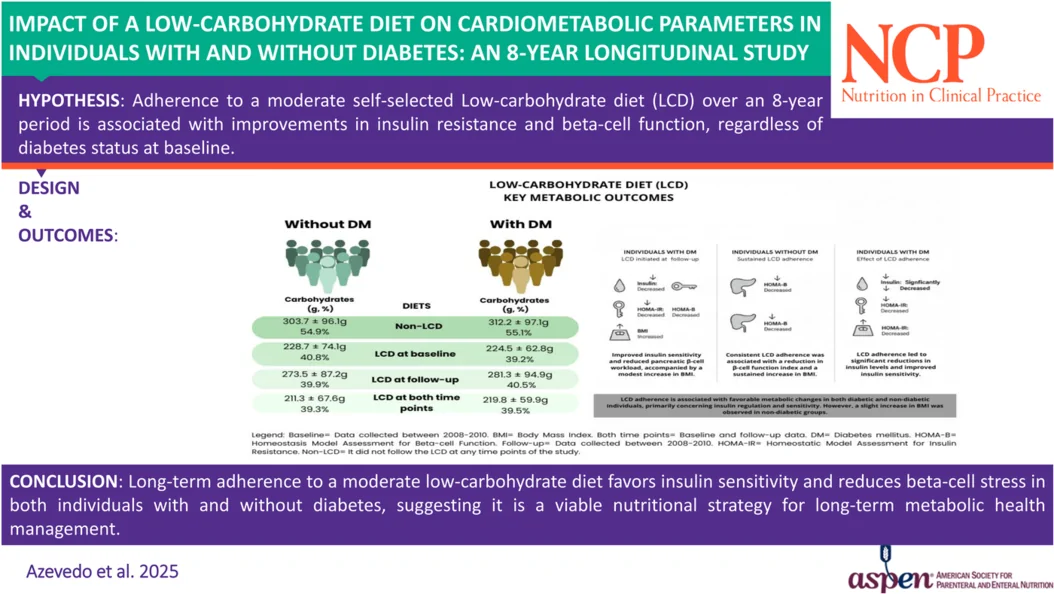

## INTRODUCTION

Chronic noncommunicable diseases (NCDs) have been leading causes of morbidity and mortality worldwide for more than two decades.^1^ Alarming data reveal that the number of adults living with diabetes globally has surpassed 800 million, more than quadrupling since 1990, with Brazil being one of the countries significantly contributing to this rise.^2^ In 2019, Brazil recorded 70,780 deaths because of diabetes, with two of every five of these deaths occurring prematurely in individuals aged 30–69 years.^3^ According to the World Health Organization (WHO), the economic burden of NCDs amounts to billions of dollars in annual losses, particularly in developing countries.^4^ In Brazil, it is estimated that the Unified Health System spent >$890 million in 2018 on hypertension, diabetes, and obesity care,^5^ with 72% of these costs attributed to individuals aged 30–69 years.

NCDs have an immediate impact on the population's quality of life. Although life expectancy is increasing worldwide, chronic diseases and high‐mortality health conditions are responsible for the highest number of disability‐adjusted life‐years lost. These conditions accounted for 100 million additional healthy years lost in 2019 compared with 2000.^6^ Lifestyle changes and dietary interventions play a crucial role in preventing and mitigating these comorbidities,^7^ given the significant role of nutrition in metabolic and physiological regulation, including the improvement of insulin sensitivity and weight management.^8^

Carbohydrate consumption is widely recognized as a contributor to the rising rates of obesity and associated cardiometabolic issues, such as diabetes and cardiovascular disease. Consequently, low‐carbohydrate diets (LCDs) have been largely studied and promoted in the media, making them a popular choice among both healthy and nonhealthy individuals. Diets with very low carbohydrate intake (up to 12% of total energy) have shown significant improvements in cardiometabolic risk factors in the short term,^9^ and recent meta‐analyses have supported these findings.^10^ However, maintaining an LCD can be challenging, especially in populations for whom carbohydrates constitute an average of 60% of total energy intake, such as in Brazil.^11^

Although the literature extensively documents the effects of prescribed LCD protocols,^8^, ^9^, ^10^, ^11^ there is limited evidence on how spontaneous, long‐term dietary patterns affect metabolic health, particularly in large population‐based samples. This study addresses this gap as, unlike clinical interventions, it examines self‐selected diets followed without a specific dietary protocol. Thus, the study aims to evaluate the impact of a self‐selected LCD on cardiometabolic parameters in individuals with and without diabetes over an 8‐year longitudinal study within the Brazilian Longitudinal Study of Adult Health (ELSA‐Brasil) cohort.

## MATERIALS AND METHODS

### Design and study participants

The present study used data obtained from the baseline (2008–2010) and 8 years of follow‐up (2017–2019) of ELSA‐Brasil, a prospective cohort study that aims to investigate the incidence and risk factors for chronic diseases, especially cardiovascular disorders and diabetes. The ELSA‐Brasil cohort includes 15,105 active and retired public employees, aged between 35 and 74 years at baseline, from five Brazilian universities and one research institute. The follow‐up had 14,017 participants, representing 92.8% follow‐up. Written informed consent was obtained from all participants and further details on the study design can be found in previous publications.^12^, ^13^, ^14^

For the present study, losses to follow‐up and deaths were excluded. Also, participants with implausible energy intake (<500 or >4000 kcal^15^), bariatric surgery, missing carbohydrate intake data, and missing outcomes at baseline and follow‐up were excluded. The final sample consisted of 9658 participants (Figure 1).

**Figure 1:**
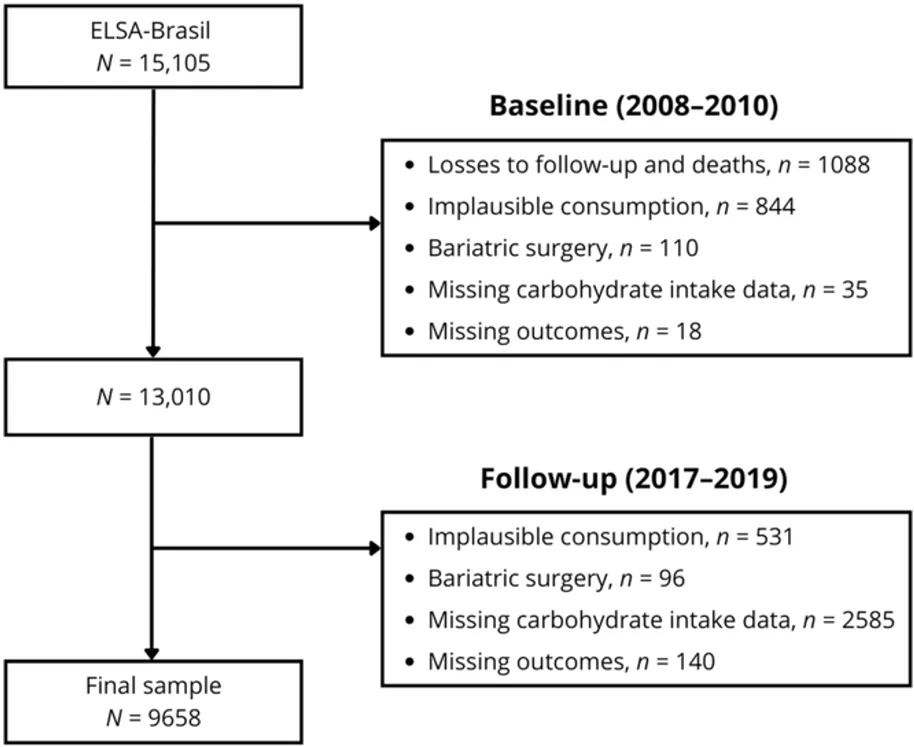
Sample flowchart. ELSA‐Brasil, Brazilian Longitudinal Study of Adult Health.

### Data collection

Food consumption, anthropometric, hemodynamic, biochemical, and lifestyle data were obtained through face‐to‐face interviews, examinations, and measurements using standardized questionnaires and well‐established protocols, conducted by trained and certified advisors. Strict quality assurance and quality control measures were adopted at all investigational centers at all time points.^13^ Blood samples were collected after 12 h of fasting, stored at −80°C, and analyzed at a single center.^16^ Medication use was assessed by asking participants about the medications they were taking continuously in the past 15 days and by checking their prescriptions and medication packages during the study visits.

### Assessment of dietary intake

Baseline dietary intake was assessed by a semiquantitative food frequency questionnaire (FFQ) with 114 food items developed and validated in the target population.^17^ Participants were asked to report the frequency and portion size of food items consumed in the past 12 months. For each item, the frequency of consumption (with eight response options ranging from “more than 3×/day” to “never/almost never”) and the number of servings consumed were assessed by the Nutrition Data System for Research software (University of Minnesota) to obtain information about macronutrients (grams/day), total energy (kilocalories), and other food items from the FFQ. More details about the FFQ have been published previously.^18^, ^19^

Food intake at follow‐up was assessed using the short version of the FFQ, which contains 76 food items and was shown to still maintain a relatively good ability to measure energy and selected nutrients.^20^

For the analysis of the present study, LCD adherence was classified as carbohydrate intake of <45% of the total energy consumed per day, following the guidelines from the Brazilian Diabetes Society.^21^ Participants adhering to an LCD were categorized into three groups: baseline (those who followed an LCD only during baseline), follow‐up (those who followed it only during the follow‐up), and both time points (those who adhered to an LCD during both time points of the study). Those individuals who consume above what is considered LCD at both time points in the study (carbohydrates ≥45% of total energy) were classified as adherents to the “Non‐LCD,” following the classification pattern of previous studies.^21^, ^22^

### Assessment of diabetes

Participants were diagnosed with diabetes at baseline based on self‐reported medical history, use of hypoglycemic medications, or specific biochemical criteria, including serum hemoglobin A1c levels >6.5% or fasting serum glucose levels >126 mg/dl.^23^

### Assessment of cardiometabolic variables (outcomes)

Biochemical parameters (basal insulin [µU/ml] and fasting plasma glucose [mg/dl]) were obtained from blood samples collected by venipuncture after an overnight fasting (12–14 h) with the collection room temperature maintained between 20°C and 24°C. The samples were properly stored at −80°C until they were transported to the project's Central Laboratory, located at the University Hospital of São Paulo. The organization of the Central Laboratory, as well as the logistics of collection and transportation of biological material at ELSA‐Brasil, have been previously described.^24^

The homeostasis model assessment for insulin resistance (HOMA‐IR) was calculated as (fasting glucose [mmol/L] × 0.0555 × fasting serum insulin [mUI/L]/22.5), and HOMA for beta cell function (HOMA‐B) was calculated as (20 × fasting insulin [µU/ml]/[fasting glucose (mmol/L) – 3.5]).^25^

Blood pressure (mm Hg) was measured after a 5‐min rest, with the participant sitting in a calm environment with controlled temperature (20°C–24°C). A validated oscillometric device (Omron HEM 705 CPINT) and cuffs appropriate for the arm circumference of each participant were used. Three measurements were obtained with a 1‐min interval between them. Casual blood pressure was considered using the average of the last two measurements.^24^

Body mass index (BMI) was calculated based on weight and height data (body weight divided by height squared [kg/m^2^]).

### Assessment of variables

During the interviews, participants reported sociodemographic, lifestyle, and health data.

#### Sociodemographic data

Age was assessed as mean and SD, self‐perception of race and skin color was recorded and characterized as “White” and “non‐White” (Brown, Black, Asian, and Indigenous). Marital status was classified as “married,” “divorced,” and “single.” Education was classified as “Incomplete elementary,” “Complete elementary,” “Complete high school,” and “University degree.” The per capita family income was calculated from the total net income of the family over the last 3 months, divided by the number of people who depend on this income to live. Subsequently, this variable was subdivided into tertiles, representing different income strata in the sample.

#### Anthropometric data

Waist circumference (WC) was measured with the participant standing upright, breathing normally, feet together, upper clothing, and arms crossed in front of the chest. The measurement was taken with an inextensible tape measure at the midpoint between the iliac crest and the lower edge of the last rib.^26^

#### Health and lifestyle

Self‐reported health status was classified as “good/very good,” “regular,” and “bad/very bad.” Smoking status was classified into three categories: “former smoker,” “current smoker,” and “never smoked.” To obtain the physical activity in metabolic equivalents (METs; min/week), the long version of the International Physical Activity Questionnaire, validated for Brazil, was used.^27^ Alcohol (grams/day), protein (grams/day), and energy (kilocalories/day) consumption data were obtained from the FFQ.

### Data analysis

Continuous variables were expressed as mean ± SD and median (IQR), and categorical variables were presented as counts and proportions (%). Participants were classified according to LCD adherence and diabetes diagnosis. The chi‐square test was used to compare proportions. The Kolmogorov‐Smirnov test was conducted to identify the normality of continuous variables. The comparison of means between independent samples was conducted using one‐way analysis of variance, followed by the Bonferroni post hoc test for pairwise comparisons when statistically significant differences were detected. Additionally, the comparison of medians was performed using the Kruskal‐Wallis test, with the Dunn‐Bonferroni post hoc test applied to identify significant differences between specific group pairs where appropriate. For the longitudinal analysis, the quantile regression test was applied to assess the association between the LCD and deltas (Δ; data difference between follow‐up and baseline), BMI, basal insulin, fasting plasma glucose, HOMA‐IR, HOMA‐B, systolic blood pressure (SBP), and diastolic blood pressure (DBP) according to diabetes diagnosis. To define the adjustment model for each outcome, bivariate analyses were conducted between the covariables and the exposure (Table 1) and outcomes (Table S1 and S2). The variables selected for inclusion in the adjustment model were those that showed statistical significance (*P* < 0.200) for both exposure and the outcome or those that were deemed plausible based on the literature. Therefore, ΔBMI was adjusted for sex, income (baseline), Δage, ΔMETs, Δtotal cholesterol, and antihypertensive medication use (baseline). ΔHOMA‐B was adjusted for sex, Δage, ΔWC, and Δenergy. ΔHOMA‐IR was adjusted for sex, Δage, Δweight, Δenergy, and antihypertensive medication use (baseline and follow‐up). ΔGlucose was adjusted for sex, income (baseline), Δage, ΔWC, and antihypertensive medication use (baseline). ΔInsulin was adjusted for antihypertensive medication use (baseline), ΔWC and Δenergy. ΔSBP and ΔDBP were adjusted for sex, Δage, ΔWC, Δtotal cholesterol, and antihypertensive medication use (baseline and follow‐up). The raw model is presented in Table S3. Data analysis was conducted using Stata 16.0 (https://www.stata.com, accessed on October 18, 2024).

## RESULTS

Among 9658 participants evaluated (mean ± SD age of 51.0 ± 8.8 years at baseline), approximately 6.1% were classified as having diabetes considering both time points of the study. Most of the individuals with diabetes were women, were non‐White, were married, and belonged to the low‐income strata. Carbohydrate intake in the non‐LCD group was 312.2 ± 97.1 g/day (55.1% of the total energy intake). Approximately 42.2% adhered to the LCD at least one time during the study. Carbohydrate intake was 224.5 ± 62.8 g/day (39.2% of the total energy intake), 281.3 ± 94.9 g/day (40.5% of the total energy intake), and 219.8 ± 59.9 g/day (39.5% of total energy intake), according to LCD adherence (only at baseline, only at follow‐up, and both time points, respectively). Regarding anthropometric and biochemical changes (Table S4), significant variations were observed in ΔWC, Δtotal cholesterol, and Δglycated hemoglobin. Concerning dietary changes (Table S4), the largest variations in energy and protein intake occurred among those who followed the LCD only at baseline. The greatest variation in carbohydrate intake was observed among those who followed the diet only at the follow‐up, being different from other comparison groups, except for those who adhered to the LCD in both time points. Regarding lipid, the most significant variation occurred among participants who followed the LCD in both time points but was not different from the group who adhered to LCD only at baseline.

Among individuals without diabetes, the mean ± SD age was 55.1 ± 8.2 years, most were women, were White, reported good/very good health status, and had never smoked. For those following a non‐LCD, the mean ± SD carbohydrate intake was 303.7 ± 96.1 g/day, representing 54.9% of total energy intake. Approxmately 45.4% adhered to the LCD at some time point of the study. Carbohydrate intake was 228.7 ± 74.1 g/day (40.8% of the total energy intake), 273.5 ± 87.2 g/day (39.9% of the total energy intake), and 211.3 ± 67.6 g/day of carbohydrate (39.3% of the total energy intake) according to LDC adherence (only at baseline, only at follow‐up, and both time points, respectively) (Table 1). Regarding anthropometric and biochemical changes (Table S4), the greatest variations in METs, WC, and Δweight occurred in the group that followed the LCD in both time points, and the greatest variations in HDL levels and total cholesterol occurred in those who followed the LCD only at baseline. Concerning dietary characteristics, among participants without diabetes, the greatest variations in energy, protein, and lipid intake occurred among participants who followed the LCD only at baseline (Table S4). As for carbohydrate intake, the greatest variation was observed among those who followed the LCD only at the follow‐up, being different from other comparison groups.

**Table 1:** Characteristics of participants stratified by the presence of DM and LCD adherence.

Variables	Without DM						With DM					
	Total	Non‐LCD	LCD			*P* value	Total	Non‐LCD	LCD			*P* value
			Baseline	Follow‐up	Both time points				Baseline	Follow‐up	Both time points	
Age, median (IQR), years	50 (44–57)	50 (44–57)	50 (45–58)	49 (44–56)	51 (45–58)	**0.014**	54 (49–61)	54 (49–61)	55.5 (49–63.5)	55 (50–60)	54 (49–59)	0.489
Sex, *n* (%)						**0.003**						0.847
Male	3721 (41.0)	2084 (42.1)	312 (37.8)	754 (38.4)	571 (42.9)		282 (47.9)	161 (47.3)	30 (53.6)	62 (67.0)	29 (47.5)	
Female	5348 (59.0)	2862 (57.9)	514 (62.2)	1211 (61.6)	761 (57.1)		307 (52.1)	179 (52.6)	26 (46.4)	70 (53.0)	32 (52.5)	
Race, *n* (%)						**<0.001**						0.069
White	5094 (56.8)	2518 (51.5)	474 (57.7)	1173 (60.4)	929 (70.3)		222 (38.1)	113 (33.7)	27 (49.1)	56 (42.4)	26 (42.6)	
Non‐White	3879 (43.2)	2371 (48.5)	347 (42.3)	769 (39.6)	392 (29.7)		361 (61.9)	222 (66.3)	28 (50.9)	76 (57.6)	35 (57.4)	
Marital status, *n* (%)						0.251						0.345
Married	5964 (65.8)	3248 (65.7)	540 (65.4)	1293 (65.3)	883 (66.3)		378 (64.2)	217 (63.8)	37 (66.1)	83 (62.9)	41 (67.2)	
Single	980 (10.8)	540 (10.9)	81 (9.8)	234 (11.9)	125 (9.4)		57 (9.7)	35 (10.3)	9 (16.1)	9 (6.8)	4 (6.6)	
Divorced	2125 (23.4)	1158 (23.4)	205 (24.8)	205 (24.8)	324 (24.3)		154 (26.2)	88 (25.9)	10 (17.8)	40 (30.3)	16 (26.2)	
Income strata, *n* (%)						**<0.001**						**<0.001**
Low	2598 (28.7)	1797 (36.4)	189 (23.0)	442 (22.6)	170 (12.8)		245 (41.6)	162 (47.7)	19 (33.9)	49 (37.1)	15 (24.6)	
Intermediary	3009 (33.3)	1714 (34.7)	261 (31.8)	667 (34.0)	367 (27.6)		190 (32.3)	114 (33.4)	16 (28.6)	39 (29.6)	21 (34.4)	
High	3439 (38.0)	1423 (28.9)	371 (45.2)	851 (43.4)	794 (59.6)		154 (26.1)	64 (18.8)	21 (37.5)	44 (33.3)	25 (41.0)	
Use of hypertensive medication (baseline), *n* (%)						0.196						0.474
Yes	2123 (23.4)	1137 (23.0)	211 (25.5)	445 (22.7)	330 (24.8)		330 (56.1)	194 (57.2)	34 (60.7)	73 (55.3)	29 (47.5)	
No	6941 (76.6)	3808 (77.0)	615 (74.5)	1519 (77.3)	999 (75.2)		258 (43.9)	145 (42.8)	22 (39.3)	59 (44.7)	32 (54.5)	
Use of hypertensive medication (follow‐up), *n* (%)						0.686						0.637
Yes	3298 (36.7)	1773 (36.2)	307 (37.7)	720 (37.0)	498 (37.6)		394 (67.6)	224 (66.7)	42 (75.0)	86 (66.2)	42 (68.9)	
No	5684 (63.3)	3126 (63.8)	507 (62.3)	1226 (63.0)	825 (62.4)		189 (32.4)	112 (33.3)	14 (25.0)	44 (33.8)	19 (31.1)	

*Note*: *P* values used the chi‐square test; values in bold are statistically significant (*P* < 0.05). Baseline data were collected between 2008 and 2010. Follow‐up data were collected between 2017 and 2019. Data for both time points were collected at baseline and at follow‐up.

Abbreviations: DM, diabetes mellitus; LCD, low‐carbohydate diet.

Table 2 presents changes in cardiometabolic parameters at 8‐year follow‐up stratified by the presence of diabetes and LCD adherence. Among participants without diabetes, the greatest variations in BMI were observed in those who followed the LCD in both time points, whereas the largest variations in HOMA‐B, HOMA‐IR, insulin, and SBP were seen in individuals who followed the LCD only at baseline. Among participants with diabetes, the greatest changes in HOMA‐IR and insulin were observed in those who followed the LCD only at baseline. Therefore, quantile regression was performed to assess cardiometabolic parameters under the effect of LCD, adjusting for confounding variables (Table 3). In the group of participants without diabetes, among individuals who initiated LCD at the follow‐up and those who followed LCD in both time points, LCD was associated with increased BMI and decreased levels of glucose, insulin, HOMA‐B, and HOMA‐IR. Additionally, a decrease in HOMA‐IR was observed in the group of participants with diabetes that adhered to an LCD in both time points.

**Table 2:** Changes in cardiometabolic parameters at 8‐year follow‐up stratified by the presence of DM and LCD adherence.

Variables	Without DM, median (IQR)						With DM, median (IQR)					
	Total	Non‐LCD	LCD			*P* value	Total	Non‐LCD	LCD			*P* value
			Baseline	Follow‐up	Both time points				Baseline	Follow‐up	Both time points	
ΔBMI, kg/m^2^	0.93 (−0.2 to 2.2)	0.88 (−0.3 to 2.1)	0.95 (−0.2 to 2.2)	1.00 (−0.2 to 2.3)	1.05 (−0.2 to 2.3)	**0.021**	−0.47 (−1.9 to 1.1)	−0.56 (−1.8 to 1.0)	−0.55 (−2.4 to 1.2)	−0.26 (−2.1 to 1.2)	−0.47 (−2.2 to 1.0)	0.858
ΔHOMA‐B	42.18 (11.8 to 74.4)	43.53 (13.1 to 75.5)	45.22 (13.3 to 74.9)	39.38 (10.3 to 71.9)	39.73 (9.0 to 73.8)	**0.009**	18.87 (−4.2 to 58.8)	20.05 (−3.1 to 59.6)	17.50 (−5.9 to 45.1)	18.16 (−5.4 to 51.3)	18.87 (−3.6 to 70.3)	0.749
ΔHOMA‐IR	1.16 (0.3 to 2.3)	1.19 (0.3 to 2.3)	1.26 (0.4 to 2.3)	1.10 (0.3 to 2.2)	1.12 (0.2 to 2.2)	**0.026**	1.51 (−0.2 to 4.2)	1.61 (−0.1 to 4.8)	2.35 (−0.2 to 5.3)	1.57 (−0.2 to 3.5)	0.77 (−1.0 to 2.9)	**0.028**
ΔInsulin, µU/ml	4.59 (1.4 to 8.3)	4.65 (1.4 to 8.3)	4.79 (1.8 to 8.4)	4.40 (1.2 to 7.8)	4.53 (1.2 to 8.2)	**0.022**	4.59 (−0.4 to 9.7)	4.85 (0.3 to 12.1)	5.46 (0.2 to 10.5)	4.68 (−1.3 to 8.1)	3.53 (−1.3 to 6.9)	**0.068**
ΔGlucose, mg/dl	−1.00 (−6.0 to 5.0)	−1.00 (−7.0 to 5.0)	0 (−6.0 to 6.0)	−1.00 (−6.0 to 5.0)	−1 (−6.0 to 5.0)	0.479	−4.00 (−34.0 to 27.0)	−2.00 (−33.0 to 29.5)	−1.00 (−33.5 to 30.5)	−5.5 (−36.5 to 22.5)	−14.00 (−57.0 to 9.0)	0.291
ΔSBP, mm Hg	2.50 (−6.0 to 11.0)	2.50 (−5.5 to 11.5)	3.50 (−6.0 to 12.0)	2.00 (−6.5 to 10.0)	3.00 (−5.0 to 10.5)	**0.020**	1.50 (−9.5 to 11.0)	1.50 (−9.5 to 10.7)	2.50 (−8.0 to 16.7)	−0.25 (−11.2 to 9.2)	0.50 (−10.0 to 13.0)	0.663
ΔDBP, mm Hg	1.00 (−5.0 to 6.5)	1.00 (−5.0 to 6.5)	0.50 (−5.5 to 7.0)	0.50 (−5.5 to6.0)	1.00 (−5.0 to 6.5)	0.283	−3.00 (−9.5 to 3.0)	−3.25 (−9.0 to 2.5)	−2.00 (−9.5 to 4.7)	−3.25 (−10.2 to 2.7)	−4.00 (−11.5 to 3.0)	0.740

*Note*: Kruskal‐Wallis test with post hoc Dunn‐Bonferroni was used. Values in bold are statistically significant (*P* < 0.05). Baseline data were collected between 2008 and 2010. Follow‐up data were collected between 2017 and 2019. Data for both time points were collected at baseline and at follow‐up.

Abbreviations: Δ, data difference between follow‐up and baseline; BMI, body mass index; DBP, diastolic blood pressure; DM, diabetes mellitus; HOMA‐B, homeostasis model assessment for beta cell function; HOMA‐IR, homeostasis model assessment for insulin resistance; LCD, low‐carbohydrate diet; SBP, systolic blood pressure.

**Table 3:** Results of final adjusted model of the quantile regression.

Variables	Adjusted model, median (95% CI)							
	Without DM				With DM			
	Non‐LCD	LCD			Non‐LCD	LCD		
		Baseline	Follow‐up	Both time points		Baseline	Follow‐up	Both time points
ΔBMI, kg/m^2^	**Ref**	0.08 (−0.1 to 0.2)	**0.16 (0.1 to 0.3)**	**0.29 (0.2 to 0.4)**	**Ref**	0.06 (−0.8 to 0.9)	0.32 (−0.3 to 0.9)	0.49 (−0.3 to 1.3)
ΔHOMA‐B	**Ref**	−1.28 (−5.1 to 2.6)	**−4.95 (−7.7 to 2.1)**	**−3.21 (−6.4 to −0.1)**	**Ref**	1.44 (−13.4 to 16.3)	−4.08 (−14.5 to 6.3)	0.93 (−13.2 to 15.1)
ΔHOMA‐IR	**Ref**	0.03 (−0.1 to 0.2)	**−0.11 (−0.2 to −0.1)**	**−0.05 (−0.2 to −0.1)**	**Ref**	0.83 (−0.2 to 1.8)	**−0.80 (−1.5 to −0.1)**	**−1.25 (−2.2 to −0.3)**
ΔInsulin, µU/ml	**Ref**	0.19 (−0.2 to 0.6)	**−0.33 (−0.6 to −0.1)**	−0.29 (−0.6 to 0.1)	**Ref**	1.51 (−1.0 to 4.0)	−1.37 (−3.2 to 0.4)	**−3.61 (−6.0 to −1.2)**
ΔGlucose, mg/dl	**Ref**	0.50 (−0.2 to 1.2)	0.27 (−0.3 to 0.8)	−0.10 (−0.7 to 0.5)	**Ref**	−1.91 (−16.1 to 12.2)	−3.20 (−13.3 to 6.8)	−9.05 (−22.8 to 4.7)
ΔSBP, mm Hg	**Ref**	0.53 (−0.6 to 1.7)	−0.60 (−1.5 to 0.2)	0.47 (−0.5 to 1.4)	**Ref**	−0.41 (−6.0 to 5.2)	−1.66 (−5.6 to 2.3)	0.30 (−5.1 to 5.7)
ΔDBP, mm Hg	**Ref**	0.25 (−0.5 to 0.9)	−0.10 (−0.6 to 0.4)	0.44 (−0.1 to 1.1)	**Ref**	0.80 (−2.3 to 3.9)	−0.14 (−2.3 to 2.1)	−0.38 (−3.3 to 2.6)

*Note*: Adjusted model: ΔBMI was adjusted for sex, income (baseline), Δage, Δmultiples of metabolic equivalents, Δtotal cholesterol, and antihypertensive medication use (baseline). ΔHOMA‐B was adjusted for sex, Δage, Δwaist circumference, and Δenergy. ΔHOMA‐IR was adjusted for sex, Δage, Δweight, Δenergy, and antihypertensive medication use (baseline and follow‐up). ΔGlucose was adjusted for sex, income (baseline), Δage, Δwaist circumference, and antihypertensive medication use (baseline). ΔInsulin was adjusted for antihypertensive medication use (baseline), Δwaist circumference, and Δenergy. ΔSBP and ΔDBP were adjusted for sex, Δage, Δwaist circumference, Δtotal cholesterol, and antihypertensive medication use (baseline and follow‐up). Baseline data were collected between 2008 and 2010. Follow‐up data were collected between 2017 and 2019. Data for both time points were collected at baseline and at follow‐up. Bold values are statistically significant.

Abbreviations: Δ, data difference between follow‐up and baseline; BMI, body mass index; DBP, diastolic blood pressure; DM, diabetes mellitus; HOMA‐B, homeostasis model assessment for beta cell function; HOMA‐IR, homeostasis model assessment for insulin resistance; LCD, low‐carbohydrate diet; Ref, reference; SBP, systolic blood pressure.

Quantile regression was performed to assess cardiometabolic parameters under the effect of the LCD (Table 3). After adjusting the model for each outcome, in the group of participants without diabetes, individuals who began following the LCD at the follow‐up or adhered to it across both time points showed an increase in BMI and a decrease in HOMA‐B and HOMA‐IR levels. Those who followed the LCD only at the follow‐up exhibited reduced glucose and insulin levels. Additionally, a decrease in both HOMA‐IR and insulin levels was observed in the group of participants with diabetes who adhered to the LCD in both time points of the study.

## DISCUSSION

Over the 8‐year follow‐up period, we observed an increase in BMI, and metabolic parameters including HOMA‐B, HOMA‐IR, and insulin levels declined among participants without diabetes who adhered to an LCD at either time point of the study. Among participants with diabetes, HOMA‐IR and insulin levels decreased specifically in those with consistent adherence to the LCD.

The prevalence of LCD adherence was 9.1% only at baseline and 21.7% only at follow‐up, and those who adhered to an LCD in both time points represented 14.4% of the sample. These values are similar to those reported in populations from the United Kingdom,^28^ Finland,^29^ the United States,^29^ and Australia,^30^ where prevalence ranges between 7% and 17%. An LCD can provide benefits to the cardiometabolic health of middle‐aged individuals, especially because it is at this stage that increased risk factors naturally tend to emerge,^31^, ^32^ such as those associated with hypertension, IR, and visceral fat accumulation,^32^ which are critical contributors to the development of chronic diseases.

In our study, following an LCD either in both time points or starting at the follow‐up was associated with an increase in BMI among participants without diabetes. Two possible explanations can be proposed. First, an increase in weight‐related parameters may encourage individuals to adopt restrictive diets; given the widespread popularity of the LCD, this could serve as a key motivation for its adoption.^29^, ^33^ Second, following an LCD may contribute to an increase in BMI because of factors related to diet composition and metabolic responses. Although LCDs are commonly associated with weight loss, the body's adaptation to a potentially excessive energy intake and the use of alternative energy sources (fats and proteins) may not necessarily facilitate weight loss in all individuals.^34^

Moreover, because the study population consisted of middle‐aged and older adults—a group predisposed to fat redistribution and accumulation because of natural physiological aging processes—the observed increase in BMI may be a consequence of these changes. Although LCDs are commonly associated with weight loss in clinical trials, spontaneous adherence in a real‐world setting may not necessarily facilitate weight reduction in all individuals.

Our study shows significant reductions in insulin levels. Most research on LCD benefits for insulin is based on ketogenic diets with 20–50 g of daily carbohydrates^35^ (10%–20% of total energy intake). Because carbohydrate intake is the strongest driver of insulin secretion,^36^ greater restriction of this macronutrient is expected to result in lower circulating insulin levels. However, the long‐term adherence to ketogenic diets poses significant challenges because of undesirable clinical and physiological effects associated with extreme carbohydrate restriction. These include a limited range of permissible foods and symptoms such as dizziness, fainting, and hypoglycemia. Consistent with our findings, a comprehensive review that included studies where carbohydrate intake was ≤40% of total energy intake demonstrated that, at a 12‐month follow‐up, LCD had positive effects on insulin concentrations^33^, ^37^ compared with baseline. These findings suggest that moderate carbohydrate restriction, as observed in our study, may provide metabolic benefits while potentially being more sustainable than stricter ketogenic approaches.

Reductions in HOMA‐B and HOMA‐IR indices were observed among individuals without diabetes who followed an LCD in both time points. The decrease in HOMA‐B suggests a reduced demand for insulin secretion by the pancreas, wich may indicate improved insulin sensitivity,^38^ thereby lowering the need for compensatory insulin production. The decrease in HOMA‐IR further supports this interpretation, reflecting enhanced insulin sensitivity—one of the primary expected benefits of diets with a lower glycemic and insulinemic load, such as an LCD.^39^ Reduced carbohydrate intake may lead to decreased glycemic stimulation, resulting in lower insulin secretion and, consequently, reduced chronic stress on pancreatic beta cells. These findings support the hypothesis that dietary patterns with a lower glycemic load may play a preventive role in the pathophysiology of insulin resistance, contributing to the long‐term preservation of pancreatic function. The observation of these changes in individuals without diabetes suggests that dietary intervention may exert a preventive or prophylactic effect on metabolic dysfunction. This evidence may have important implications for public health policies, particularly considering the increasing global prevalence of IR and prediabetes.

Among participants with diabetes, reductions in HOMA‐IR and insulin levels were observed, indicating improved IR in this group, despite no apparent change in beta cell function. This difference may be attributable to the sample size, but it may also reflect established pathophysiological alterations in these individuals, in whom beta cell dysfunction may be more advanced or less reversible,^25^, ^38^, ^39^ thus limiting the potential for functional recovery even in response to dietary interventions. Nonetheless, the improvement in HOMA‐IR represents a clinically relevant outcome, suggesting that an LCD may confer metabolic benefits for individuals with diabetes.^40^ It is important to note that an LCD is based on the individual's total energy expenditure. Therefore, these diets will be more effective when prescribed individually by a nutritionist, the professional responsible for providing personalized eating plans, because these diets alter the proportions of macronutrients ingested, which can lead to lack of success in achieving the proposed objective and undesirable health effects,^40^ from tiredness, fatigue, nausea, and headaches to weight gain and micronutrient deficiencies.

This study has some limitations. Dietary intake was assessed using an FFQ, which relies on self‐report and may introduce recall bias or reporting errors. However, it is important to mention that this is a validated instrument, and the use of a portion size visualization kit helps minimize the chances of bias. Additionally, the rigorous quality control applied across all study time points, particularly in biochemical, anthropometric, and dietary assessments, underscores the ELSA‐Brasil cohort's commitment to minimizing potential biases. The study population, consisting of public employees in Brazil, may limit the generalizability of the findings to other cultural or socioeconomic contexts. Additionally, it did not evaluate other dietary factors, such as the types of fats and proteins consumed. However, the study's strengths include its 8‐year longitudinal design, a high follow‐up rate (92.8%), and comprehensive data collection from the large, representative ELSA‐Brasil cohort, providing valuable insights into the long‐term effects of an LCD on cardiometabolic health. By including participants both with and without diabetes, the study broadens the understanding of LCD's impact, particularly in middle‐aged and older adult populations at substantial risk for chronic diseases.

## CONCLUSION

This 8‐year analysis of the ELSA‐Brasil cohort demonstrates that adherence to an LCD is associated with improved metabolic health. Among individuals without diabetes, there was evidence of reductions in insulin levels, HOMA‐IR, and HOMA‐B. Although LCD adherence was linked to a modest increase in BMI, participants with diabetes exhibited significant reductions in HOMA‐IR and insulin, suggesting enhanced insulin sensitivity. Notably, metabolic benefits were observed even with a 5% reduction in carbohydrate intake below conventional recommendations. These findings support the potential of moderate LCDs as a sustainable strategy for long‐term metabolic improvement in middle‐aged and older adults. Our results underscore the importance of personalized dietary approaches to maximize clinical benefits, positioning the LCD as a practical and scalable tool for metabolic risk management in real‐world settings.

## AUTHOR CONTRIBUTIONS

Maria del Carmen Bisi Molina, Leticia Batista de Azevedo, and Oscar Geovanny Enriquez‐Martinez equally contributed to the conception and design of the research. Leticia Batista de Azevedo, Maria del Carmen Bisi Molina, and Haysla Xavier Martins contributed to the analysis and interpretation of the data. Leticia Batista de Azevedo drafted the manuscript. All authors critically revised the manuscript, agree to be fully accountable for ensuring the integrity and accuracy of the work, and read and approved the final manuscript.

## CONFLICT OF INTEREST STATEMENT

None declared.

## Supporting information

Supplementary Tables 05 01 26.
