# Supplementary material for: Impact of a low‐carbohydrate diet on cardiometabolic parameters in individuals with and without diabetes: An 8‐year longitudinal study within the ELSA‐Brasil cohort
Source: Nutr Clin Pract. 2026 Feb 6;41(5):1349–59. doi: 10.1002/ncp.70099 (PMC13622392; doi:10.1002/ncp.70099)
Supplement: Supplementary file 1 [file NCP-41-1349-s001.docx]

| Supplementary Table 1: Characteristics of participants without diabetes according to outcomes variables. | | | | | | | | | | | | | | |
| --- | --- | --- | --- | --- | --- | --- | --- | --- | --- | --- | --- | --- | --- | --- |
| **Variables** | **Without DM** | | | | | | | | | | | | | |
|  | **ΔBMI (kg/m²)** | **p-value** | **ΔHOMA-B** | **p-value** | **ΔHOMA-IR** | **p-value** | **ΔInsulin (IU)** | **p-value** | **ΔGlucose (mg/dL)** | **p-value** | **ΔSBP (mmHg)** | **p-value** | **ΔDBP (mmHg)** | **p-value** |
| **Sex** |  | **<0.001** |  | **<0.001** |  | 0.440 |  | **0.007** |  | 0.091 |  | **<0.001** |  | 0.093 |
| Male | 0.70 (-0.4 , 1.7) |  | 37.34 (6.4 , 70.3) |  | 1.16 (0.2 , 2.3) |  | 4.43 (1.0 , 8.3) |  | -1.00 (-7.0 , 5.0) |  | 1.50 (-7.0 , 10.0) |  | 0.50 (-5.5 , 6.5) |  |
| Female | 1.13 (-0.1 , 2.5) |  | 45.39 (15.7 , 77.5) |  | 1.16 (0.3 , 2.1) |  | 4.71 (1.6 , 8.2) |  | -1.00 (-6.0 , 5.0) |  | 3.50 (-5.0 , 11.5) |  | 1.00 (-5.0 , 6.5) |  |
| **Race/color skin** |  | **0.015** |  | **<0.001** |  | **<0.001** |  | **<0.001** |  | **0.122** |  | 0.641 |  | **0.024** |
| White | 0.90 (-0.2 , 2.1) |  | 40.07 (10.1 , 72.2) |  | 2.50 (-5.5 , 10.5) |  | 4.32 (1.1 , 8.0) |  | -1.00 (-6.0 , 5.0) |  | 2.50 (-5.5 , 10.5) |  | 1.00 (-4.5 , 6.5) |  |
| Non-White | 0.99 (-0.2 , 2.2) |  | 44.72 (13.9 , 77.8) |  | 1.27 (0.3 , 2.3) |  | 4.87 (1.6 , 8.5) |  | -1.00 (-7.0 , 6.0) |  | 2.50 (-6.0 , 11.5) |  | 0.50 (-6.0 , 6.5) |  |
| **Marital status** |  | **<0.001** |  | **0.011** |  | 0.596 |  | 0.304 |  | 0.785 |  | 0.413 |  | 0.307 |
| Married | 0.86 (-00.2 , 2.1) |  | 41.10 (10.8 , 73.6) |  | 1.16 (0.3 , 2.2) |  | 4.52 (1.3 , 8.2) |  | -1.00 (-6.0 , 5.0)' |  | 2.50 (-5.5 , 10.5) |  | 1.00 (-5.0 , 6.5) |  |
| Single | 1.12 (-0.1 , 2.3) |  | 45.6 (17.1 , 77.2) |  | 1.21 (0.3 , 2.1) |  | 4.94 (1.5 , 8.2) |  | -1.00 (-6.0 , 5.0) |  | 2.50 (-7.0 , 10.5) |  | 0.50 (-6.0 , 6.0) |  |
| Divorced | 1.04 (-0.2 , 2.3) |  | 43.63 (13.9 , 75.4) |  | 1.61 (0.3 , 2.3) |  |  |  | -1.00 (-6.0 , 6.0) |  | 3.00 (-6.0 , 12.0) |  | 0.50 (-5.5 , 6.5) |  |
| **Income strata** |  | **<0.001** |  | **<0.001** |  | **<0.001** |  | **<0.001** |  | 0.074 |  | **<0.001** |  | **<0.001** |
| Low | 1.13 (-0.1 , 2.3) |  | 48.01 (17.3 , 84.4) |  | 1.43 (0.5 , 2.5) |  | 5.48 (2.2 , 9.5) |  | -1.00 (-6.0 , 6.0) |  | 3.50 (-5.5 , 12.5) |  | 1.00 (-5.0 , 7.0) |  |
| Intermediary | 0.98 (-0.2 , 2.2) |  | 44.18 (14.1 , 76.2) |  | 1.19 (0.3 , 2.2) |  | 4.70 (1.5 , 8.3) |  | -1.00 (-7.0 , 5.0) |  | 2.50 (-5.5 , 11.0) |  | 1.00 (-5.0 , 6.5) |  |
| High | 0.74 (-0.4 , 1.9) |  | 36.13 (6.0 , 67.3) |  | 0.97 (0.2 , 1.9) |  | 4.62 (1.4 , 8.3) |  | -1.00 (-6.0 , 6.0) |  | 2.00 (-6.5 , 10.0) |  | 0.50 (-5.5 , 5.5) |  |
| **Use of hypertensive medication (baseline)** | | **<0.001** |  | **<0.001** |  | **<0.001** |  | 0.055 |  | **<0.001** |  | **<0.001** |  | **<0.001** |
| Yes | 0.59 (-0.7 , 1.9) |  | 38.89 (5.9 , 73.6) |  | 1.35 (0.2 , 2.7) |  | 4.88 (0.9 , 9.3) |  | 0 (-7.0 , 8.0) |  | 1.50 (-10.5 , 12.0) |  | -2.00 (-9.0 , 5.0) |  |
| No | 1.03 (-0.1 , 2.2) |  | 43.27 (13.6 , 74.8) |  | 1.12 (0.3 , 2.1) |  | 4.52 (1.5 , 8.0) |  | -1.00 (-6.0 , 5.0) |  | 2.50 (-5.0 , 10.5) |  | 1.50 (-4.0 , 6.5) |  |
| **Use of hypertensive medication (follow-up)** | | **<0.001** |  | 0.457 |  | **<0.001** |  | **<0.001** |  | **<0.001** |  | **<0.001** |  | **<0.001** |
| Yes | 0.82 (-0.5 , 2.1) |  | 42.67 (10.3 , 77.6) |  | 1.42 (0.3 , 2.7) |  | 5.25 (1.6 , 9.5) |  | 0 (-6.0 , 7.0) |  | -0.50 (-11.5 , 10.0) |  | -3.00 (-9.5 , 4.0) |  |
| No | 1.01 (-0.1 , 2.2) |  | 41.56 (12.7 , 72.5) |  | 1.05 (0.2 , 1.9) |  | 4.29 (1.3 , 7.6)0 |  | -1.00 (-6.0 , 4.0) |  | 4.00 (-3.5 , 11.5) |  | 2.50 (-3.0 , 7.5) |  |
| **ΔAge (years)*** | 0.077 | **0.001** | 0.245 | **<0.001** | 0.180 | **<0.001** | 0.205 | **<0.001** | -0.067 | **<0.001** | 0.037 | **<0.001** | 0.026 | **0.011** |
| **ΔMETs (min/week)*** | 0.091 | **<0.001** | 0.042 | **<0.001** | 0.065 | **<0.001** | 0.058 | **<0.001** | 0.020 | 0.057 | 0.019 | 0.061 | 0.051 | **<0.001** |
| **ΔWeight (kg)*** | 0.979 | **<0.001** | 0.330 | **<0.001** | 0.368 | **<0.001** | 0.390 | **<0.001** | 0.198 | **<0.001** | 0.178 | **<0.001** | 0.264 | **<0.001** |
| **ΔWC (cm)** | 0.775 | **<0.001** | 0.274 | **<0.001** | 0.339 | **<0.001** | 0.346 | **<0.001** | 0.200 | **<0.001** | 0.149 | **<0.001** | 0.236 | **<0.001** |
| **ΔHC (cm)*** | 0.775 | **0.001** | 0.228 | **<0.001** | 0.252 | **<0.001** | 0.270 | **<0.001** | 0.157 | **<0.001** | 0.144 | **<0.001** | 0.212 | **<0.001** |
| **ΔHDL (mg/dL)*** | -0.230 | **<0.001** | -0.097 | **<0.001** | -0.161 | **<0.001** | -0.151 | **<0.001** | -0.101 | **<0.001** | 0.041 | **<0.001** | 0.025 | **0.014** |
| **ΔTG (mg/dL)*** | 0.267 | **<0.001** | 0.141 | **<0.001** | 0.204 | **<0.001** | 0.193 | **<0.001** | 0.140 | **<0.001** | 0.104 | **<0.001** | 0.181 | **<0.001** |
| **ΔLDL (mg/dL)*** | 0.160 | **<0.001** | 0.021 | **0.044** | -0.004 | 0.639 | 0.003 | 0.734 | 0.032 | **0.002** | 0.086 | **<0.001** | 0.168 | **<0.001** |
| **ΔTotal cholesterol (mg/dL)*** | 0.148 | **<0.001** | 0.033 | **0.001** | 0.017 | 0.098 | 0.021 | **0.039** | 0.048 | **<0.001** | 0.119 | **<0.001** | 0.209 | **<0.001** |
| **ΔGlycated HB (%)*** | 0.456 | **<0.001** | -0.097 | **<0.001** | 0.114 | **<0.001** | 0.044 | **<0.001** | 0.221 | **<0.001** | -0.008 | 0.402 | -0.004 | 0.644 |
| **ΔCalories (kcal)*** | 0.005 | 0.581 | 0.040 | **<0.001** | 0.051 | **<0.001** | 0.050 | **<0.001** | 0.018 | 0.082 | 0.029 | **0.004** | 0.004 | 0.660 |
| **ΔCarbohydrate (g)*** | -0.002 | 0.830 | 0.039 | **<0.001** | 0.051 | **<0.001** | 0.051 | **<0.001** | 0.010 | 0.303 | 0.038 | **<0.001** | 0.013 | 0.195 |
| **ΔProtein (g)*** | 0.001 | 0.973 | 0.023 | **0.027** | 0.025 | **<0.001** | 0.025 | **0.013** | 0.000 | 0.997 | -0.004 | 0.685 | -0.018 | 0.087 |
| **ΔLipid (g)*** | -0.086 | **<0.001** | -0.005 | 0.631 | -0.010 | 0.311 | -0.011 | 0.275 | -0.021 | **0.039** | 0.040 | **<0.001** | -0.040 | **<0.001** |
| **ΔAlcohol (g)*** | 0.052 | **<0.001** | 0.002 | 0.786 | 0.012 | 0.242 | 0.007 | 0.467 | 0.032 | **0.001** | 0.032 | **0.001** | 0.063 | **<0.001** |
| Data expressed as median (interquartile range) or correlation coefficients. *Spearman test. Values in bold are statistically significant (p<0.05). Δ = Data difference between follow-up and baseline. BMI = body mass index. DBP = Diastolic blood pressure. DM= Diabetes mellitus. Glycated HB= Glycated hemoglobin. HC= Hip circumference. HDL=High-Density Lipoprotein. HOMA-B = homeostasis model assessment for Beta-cell function. HOMA-IR = homeostasis model assessment for insulin resistance. LCD = Low-Carbohydrate Diet. LDL = Low-Density Lipoprotein. METs = multiples of metabolic equivalents. TG= Triglycerides. | | | | | | | | | | | | | | |

| Supplementary Table 2: Characteristics of participants with diabetes according to outcomes variables. | | | | | | | | | | | | | | |
| --- | --- | --- | --- | --- | --- | --- | --- | --- | --- | --- | --- | --- | --- | --- |
| **Variables** | **With DM** | | | | | | | | | | | | | |
|  | **ΔBMI (kg/m²)** | **p-value** | **ΔHOMA-B** | **p-value** | **ΔHOMA-IR** | **p-value** | **ΔInsulin (IU)** | **p-value** | **ΔGlucose (mg/dL)** | **p-value** | **ΔSBP (mmHg)** | **p-value** | **ΔDBP (mmHg)** | **p-value** |
| **Sex** |  | 0.687 |  | **0.032** |  | 0.528 |  | 0.104 |  | 0.649 |  | 0.833 |  | 0.558 |
| Male | -0.48 (-1.8 , 1.0) |  | 14.64 (-7.5 , 47.6) |  | 1.44 (-0.3 , 4.3) |  | 4.08 (-0.9 , 9.5) |  | -6.50 (-44.0 , 31.0) |  | 1.50 (-10.0 , 11.0) |  | -3.00 (-9.0 , 3.0) |  |
| Female | -0.47 (-2.1 , 1.1) |  | 22.50 (-0.2 , 61.3) |  | 1.63 (0.1 , 4.2) |  | 5.14 (0.2 , 9.8) |  | -3.00 (-28.0 , 19.0) |  | 1.50 (-9.5 , 11.5) |  | -3.00 (-10.0 , 2.5) |  |
| **Race/color skin** |  | 0.362 |  | 0.262 |  | **<0.001** |  | **<0.001** |  | 0.164 |  | 0.158 |  | 0.111 |
| White | -0.53 (-2.3 , 1.1) |  | 18.04 (-10.5 , 53.2) |  | 0.94 (-1.3 , 3.8) |  | 3.33 (-2.1 , 8.1) |  | -6.50 (-40.0 , 21.0) |  | 3.00 (-10.0 , 14.0) |  | -2.50 (-9.0 , 4.5) |  |
| Non-White | -0.41 (-1.8 , 1.0) |  | 19.47 (-0.2 , 58.2) |  | 1.87 (0.4 , 4.6) |  | 5.14 (0.6 , 11.4) |  | -3.00 (-30.0 , 30.0) |  | 1.00 (-9.5 , 9.5) |  | -3.50 (-10.0 , 2.5) |  |
| **Marital status** |  | 0.496 |  | 0.651 |  | 0.265 |  | 0.161 |  | 0.707 |  | 0.755 |  | 0.902 |
| Married | -0.51 (-1.9 , 1.0) |  | 18.61 (-4.4 , 57.6) |  | 1.35 (-0.2 , 3.9) |  | 4.12 (-0.4 , 9.4) |  | -7.00 (-37.0 , 26.0) |  | 1.00 (-9.0 , 9.5) |  | -3.25 (-9.5 , 3.0) |  |
| Single | -0.14 (-1.7 , 1.5) |  | 17.34 (-5.5 , 56.8) |  | 1.25 ('-0.6 , 4.2) |  | 4.01 (-1.4 , 8.4) |  | 0 (-33.0 , 13.0) |  | 2.00 (-10.0 , 8.5) |  | -2.00 (-9.5 , 2.0) |  |
| Divorced | -0.49 (-2.1 , 1.0) |  | 20.77 (-0.2 ,54.4) |  | 1.79 (0.1 , 4.8) |  | 5.72 (0.4 , 12.0) |  | -2.50 (-28.0 , 30.0) |  | 3.00 (-12.0 , 15.5) |  | -3.75 (-10.5 , 4.0) |  |
| **Income strata** |  | **0.042** |  | 0.439 |  | **<0.001** |  | **0.001** |  | **0.027** |  | 0.663 |  | 0.611 |
| Low | -0.24 (-1.4 , 1.2) |  | 17.18 (-6.1 , 52.6) |  | 2.27 (0.4 , 4.9) |  | 5.21 (0.7 , 12.0) |  | -2.00 (-33.0 , 37.0) |  | 2.50 (-9.5 , 10.5) |  | -3.00 (-10.0 , 3.5) |  |
| Intermediary | -0.71 (-2.2 , 1.1) |  | 20.77 (0.1 , 55.5) |  | 1.73 (0.2 , 4.2) |  | 4.88 (0.5 , 10.0) |  | -1.00 (-28.0 , 25.0) |  | 1.25 (-9.0 , 12.5) |  | -2.50 (-9.5 , 3.0) |  |
| High | -0.55 (-2.3 , 0.8) |  | 20.19 (-6.6 , 63.9) |  | 0.58 (-1.5 , 2.8) |  | 2.49 (-3.1 , 8.0) |  | -13.0 (-43.0 , 12.0) |  | 0.25 (-10.5 , 11.5) |  | -4.75 (-9.5 , 2.0) |  |
| **Use of hypertensive medication (baseline)** | | **0.013** |  | 0.884 |  | 0.173 |  | 0.329 |  | 0.246 |  | 0.788 |  | 0.386 |
| Yes | -0.59 (-2.2 , 0.9) |  | 18.99 (-5.3 , 58.2) |  | 1.30 (-0.3 , 4.3) |  | 4.23 (-0.9 , 9.8) |  | -7.00 (-42.0 , 27.0) |  | 1.00 (-12.0 , 13.5) |  | -3.50 (-10.0 , 2.5) |  |
| No | -0.22 (-1.5 , 1.3) |  | 18.89 (-3.1 , 55.5) |  | 1.66 (0.1 , 4.2) |  | 4.81 (0.3 , 9.5) |  | -2.50 (-30.0 , 28.0) |  | 2.00 (-7.0 , 9.5) |  | -3.00 (-9.0 , 3.5) |  |
| **Use of hypertensive medication (follow-up)** | | 0.289 |  | **0.048** |  | 0.971 |  | 0.267 |  | **0.030** |  | **0.062** |  | **<0.001** |
| Yes | -0.26 (-1.9 , 1.0) |  | 21.23 (-2.4 , 61.4) |  | 1.44 (-0.3 , 4.4) |  | 4.89 (-0.4 , 11.4) |  | -8.00 (-40.0 , 25.0) |  | 0.50 (-12.0 , 10.5) |  | -4.50 (-11.5 , 2.0) |  |
| No | -0.77 (-2.1 , 1.0) |  | 14.26 (-5.4 , 48.5) |  | 1.60 (0.1 , 3.4) |  | 4.13 (-0.3 , 8.0) |  | -1.00 (-21.0 , 29.0) |  | 3.50 (-6.0 , 11.5) |  | -1.00 (-7.5 , 4.5) |  |
| **ΔAge (years)*** | -0.013 | 0.751 | -0.004 | 0.922 | 0.098 | **0.017** | 0.060 | 0.140 | 0.085 | **0.037** | 0.005 | 0.901 | -0.039 | 0.336 |
| **ΔMETs (min/week)*** | -0.03 | 0.406 | -0.001 | 0.993 | 0.056 | 0.173 | 0.027 | 0.500 | 0.018 | 0.658 | -0.023 | 0.563 | -0.006 | 0.869 |
| **ΔWeight (kg)*** | 0.976 | **<0.001** | 0.332 | **<0.001** | 0.380 | **<0.001** | 0.447 | **<0.001** | -0.015 | 0.707 | 0.206 | **<0.001** | 0.179 | **<0.001** |
| **ΔWC (cm)** | 0.777 | **<0.001** | 0.281 | **<0.001** | 0.362 | **<0.001** | 0.408 | **<0.001** | 0.028 | 0.491 | 0.192 | **<0.001** | 0.175 | **<0.001** |
| **ΔHC (cm)*** | 0.795 | **<0.001** | 0.249 | **<0.001** | 0.259 | **<0.001** | 0.322 | **<0.001** | -0.046 | 0.259 | 0.167 | **<0.001** | 0.159 | **<0.001** |
| **ΔHDL (mg/dL)*** | -0.109 | **0.007** | -0.042 | 0.300 | -0.143 | **<0.001** | -0.160 | **<0.001** | -0.007 | 0.863 | 0.044 | 0.285 | 0.048 | 0.241 |
| **ΔTG (mg/dL)*** | 0.072 | **0.080** | -0.089 | 0.301 | 0.179 | **<0.001** | 0.057 | 0.161 | 0.318 | **<0.001** | 0.063 | 0.126 | 0.194 | **<0.001** |
| **ΔLDL (mg/dL)*** | 0.008 | 0.831 | -0.045 | 0.266 | 0.072 | 0.077 | 0.002 | 0.949 | 0.150 | **<0.001** | 0.094 | **0.022** | 0.172 | **<0.001** |
| **ΔTotal cholesterol (mg/dL)*** | -0.001 | 0.966 | -0.094 | **0.021** | 0.110 | **0.007** | -0.003 | 0.940 | 0.248 | **<0.001** | 0.128 | **<0.001** | 0.229 | **<0.001** |
| **ΔGlycated HB (%)*** | -0.137 | **<0.001** | -0.365 | **<0.001** | 0.239 | **<0.001** | -0.048 | 0.243 | 0.552 | **<0.001** | 0.061 | 0.137 | 0.091 | **0.028** |
| **ΔCalories (kcal)*** | 0.042 | 0.307 | 0.048 | 0.236 | 0.078 | 0.058 | 0.072 | 0.078 | -0.026 | 0.525 | -0.048 | 0.239 | -0.020 | 0.620 |
| **ΔCarbohydrate (g)*** | -0.020 | 0.614 | 0.032 | 0.434 | 0.078 | 0.056 | 0.081 | **0.047** | -0.011 | 0.785 | -0.032 | 0.433 | 0.003 | 0.931 |
| **ΔProtein (g)*** | 0.044 | 0.284 | 0.502 | 0.223 | 0.065 | 0.114 | 0.063 | 0.123 | -0.021 | 0.609 | -0.06 | 0.131 | -0.053 | 0.194 |
| **ΔLipid (g)*** | 0.019 | 0.640 | 0.106 | **0.010** | -0.001 | 0.963 | 0.026 | 0.519 | -0.121 | **0.003** | 0.013 | 0.749 | -0.040 | 0.326 |
| **ΔAlcohol (g)*** | 0.070 | 0.085 | 0.009 | 0.817 | -0.026 | 0.528 | -0.034 | 0.402 | 0.013 | 0.741 | 0.034 | 0.398 | 0.047 | 0.250 |
| Data expressed as median (interquartile range) or correlation coefficients. *Spearman test. Values in bold are statistically significant (p<0.05). Δ = Data difference between follow-up and baseline. BMI = body mass index. DBP = Diastolic blood pressure. DM= Diabetes mellitus. Glycated HB= Glycated hemoglobin. HC= Hip circumference. HDL=High-Density Lipoprotein. HOMA-B = homeostasis model assessment for Beta-cell function. HOMA-IR = homeostasis model assessment for insulin resistance. LCD = Low-Carbohydrate Diet. LDL = Low-Density Lipoprotein. METs = multiples of metabolic equivalents. TG= Triglycerides. | | | | | | | | | | | | | | |

| **Supplementary Table 3**. Results of the raw model of the quantile regression. | | | | | | | | |
| --- | --- | --- | --- | --- | --- | --- | --- | --- |
| **Variables** | **Raw model** | | | | | | | |
|  | **Without DM** | | | | **With DM** | | | |
|  | **Non-LCD** | **LCD** | | | **Non-LCD** | **LCD** | | |
|  |  | **Baseline** | **Follow-up** | **Both time points** |  | **Baseline** | **Follow-up** | **Both time points** |
| **ΔBMI (kg/m²)** | **ref** | 0.06 (-0.1 , 0.2) | **0.11 (0.1 , 0.2)** | **0.16 (0.1 , 0.3)** | **ref** | -0.01 (-0.1 , 0.8) | 0.29 (-0.3 , 0.9) | 0.08 (-0.7 , 0.9) |
| **ΔHOMA-B** | **ref** | 1.67 (-2.5 , 5.9) | **-4.17 (-7.2 , -1.1)** | -3.80 (-7.2 , 5.9) | **ref** | -2.84 (-18.2 , 12.5) | -1.65 (-12.6 , 9.3) | -1.15 (-16.0 , 13.7) |
| **ΔHOMA-IR** | **ref** | 0.07 (-0.1 , 0.2) | **-0.09 (-0.2 , -0.1)** | -0.06 (-0.2 , 0.1) | **ref** | 0.74 (-0.4 , 1.9) | -0.04 (-0.9 , 0.8) | -0.85 (-2.0 , 0.2) |
| **ΔInsulin (µU/mL)** | **ref** | 0.22 (-0.3 , 0.6) | 0.16 (-0.5 , 0.1) | 0.18 (-0.5 , 0.2) | **ref** | 1.27 (-1.7 , 3.2) | 0.90 (-1.9 , 1.6) | 1.23 (-3.7 , 1.1) |
| **ΔGlucose (mg/dL)** | **ref** | 0.40 (0.2 , 1.8) | 0.28 (-0.5 , 0.5) | 0.33 (-0.6 , 0.6) | **ref** | 6.09 (-9.9 , 13.9) | 4.33 (-12.5 , 4.5) | **5.87 (-23.5 , -0.5)** |
| **ΔSBP (mmHg)** | **ref** | 0.59 (-0.2 , 2.1) | 0.41 (-1.3 , 0.3) | 0.48 (-0.4 , 1.4) | **ref** | 2.75 (-3.9 , 6.9) | 1.95 (-5.3 , 2.3) | 2.65 (-6.2 , 4.2) |
| **ΔDBP (mmHg)** | **ref** | 0.38 (-1.2 , 0.2) | 0.27 (-1.0 , 0.1) | 0.31 (-0.6 , 0.6) | **ref** | 1.69 (-1.8 , 4.8) | 1.20 (-1.8 , 2.8) | 1.63 (-3.7 , 2.7) |
| Data expressed as median (95% Confidence Interval). Δ = Data difference between follow-up and baseline. Baseline = Data collected between 2008-2010. Both time points = Baseline and follow-up data. BMI = body mass index. DBP = Diastolic blood pressure. HOMA-B = homeostasis model assessment for Beta-cell function. HOMA-IR = homeostasis model assessment for insulin resistance. LCD = Low-Carbohydrate Diet. Ref = reference category. SBP = Systolic blood pressure. | | | | | | | | |

| Supplementary Table 4. Characteristics of participants stratified by the presence of diabetes and low-carbohydrate adherence. | | | | | |  |  |  |  |  |  |  |  |
| --- | --- | --- | --- | --- | --- | --- | --- | --- | --- | --- | --- | --- | --- |
| **Variables** | **Without diabetes** | | | | | | | **With diabetes** | | | | | |
|  | **Total** | **Non-LCD** | **LCD** | | | | **p-value** | **Total** | **Non-LCD** | **LCD** | | | **p-value** |
|  |  |  | **Baseline** | **Follow-up** | **Both time points** | |  |  |  | **Baseline** | **Follow-up** | **Both time points** |  |
| **Anthropometric and Biochemical Changes** | | | | | | | | | | | | | |
| **ΔMETs (min/week)** | 0 (-657.0 , 82.5) | 0 (-594.0 , 0) | 0 (-684.0 , 114.0) | 0 (-720.0 , 66.0) | 0 (-720.0 , 198.0) | | 0.408 | 0 (-495.0 , 132.0) | 0 (-678.0 , 7.5) | 0 (-396.0 , 91.5) | 0 (-333.2 , 300.5) | 0 (-480.0 , 256.5) | 0.143 |
| **ΔWeight (kg)** | 2.15 (-1.1 , 5.4) | 2.00 (-1.2 , 5.2) | 2.25 (-1.2 , 5.4) | 2.30 (-0.9 , 5.8) | 2.55 (-0.8 , 5.9) | | **0.002** | -1.65 (-5.7 , 2.3) | -2.00 (-5.5 , 2.1) | -1.45 (-6.8 , 1.8) | -0.85 (-5.3 , 2.8) | -1.05 (-6.8 , 3.1) | 0.638 |
| **ΔWC (cm)** | 5 (1.3 , 8.9) | 5.00 (1.2 , 8.7) | 5.20 (1.5 , 9.1) | 5.20 (1.4 , 8.9) | 5.30 (1.5 , 9.4) | | 0.214 | 2.35 (-1.7 , 6.7) | 2.00 (-1.7 , 6.8) | 2.60 (-2.1 , 7.5) | 2.95 (-1.6 , 6.9) | 2.90 (-2.2 , 5.7) | 0.854 |
| **ΔHC (cm)** | 2.10 (-0.4 , 4.9) | 2.10 (-0.5 , 4.8) | 2.10 (-0.7 , 4.9) | 2.20 (-0.3 , 5.0) | 2.30 (-0.6 , 5.2) | | 0.306 | -0.20 (-3.3 , 2.5) | -0.20 (-3.3 , 2.2) | 0.85 (-1.8 , 2.9) | -0.20 (-3.2 , 3.0) | -0.60 (-4.0 , 1.9) | 0.442 |
| **ΔHDL (mg/dL)** | -2 (-8.0 , 4.0) | -2.00 (-8.0 , 4.0) | -3.00 (-8.0 , 4.0) | -1.00 (-8.0 , 5.0) | -2.00 (-8.0 , 4.0) | | **<0.001** | -1 (-6.0 , 5.0) | -1.00 (-6.0 , 6.0) | 0 (-4.0 , 6.0) | -2.00 (-8.0 , 3.5) | -1.00 (-6.0 , 7.0) | 0.215 |
| **ΔTG (mg/dL)** | 7 (-22.0 , 33.0) | 6.00 (-23.0 , 34.0) | 9.00 (-21.0 , 36.0) | 7.00 (-21.0 , 33.0) | 7.00 (-22.0 (33.0) | | 0.460 | -13 (-61.5 , 25.0) | -13.00 (-56.0 , 23.0) | -13.00 (-50.5 , 31.5) | -12.00 (-73.5 , 26.5) | -14.00 (-70.0 , 27.0) | 0.641 |
| **ΔLDL (mg/dL)** | -12 (-33.0 , 7.0) | -12.00 (-33.0 , 6.0) | -11.00 (-33.0 , 7.0) | -11.00 (-32.0 , -8.0) | -12.00 (-35.0 , 6.0) | | 0.202 | -17 (-43.0 , 2.0) | -17.50 (-43.0 , 1.0) | -10.50 (-39.5 , 7.5) | -19.50 (-43.0 , 1.0) | -16.00 (-42.0 , 3.0) | 0.600 |
| **ΔTotal cholesterol (mg/dL)** | -12 (-37.0 , 9.0) | -12.00 (-37.0 , 9.0) | -13.00 (-36.0 , 11.0) | -10.00 (-35.0 , 11.0) | -12.00 (-40.0 , 8.0) | | **0.040** | -20.50 (-53.5 , 3.0) | -21.00 (-51.0 , 1.0) | -6.00 (-54.0 , 21.5) | -21.50 (-59.0 , 3.0) | -23.00 (-46.0 , -1.0) | 0.256 |
| **ΔGlycated HB (%)** | 0.30 (0 , 0.7) | 0.30 (0 , 0.7) | 0.30 (-0.1 , 0.7) | 0.30 (0 , 0.6) | 0.30 (-0.1 , 0.6) | | **0.043** | -0.30 (-1.1 , 0.9) | -0.30 (-1.1 , 0.9) | 0 (-0.8 , 1.2) | -0.30 (-1.1 , 1.2) | -0.70 (-1.0 , 0.4) | 0.220 |
| **Dietary Changes** | | | | | | | | | | | | | |
| **ΔCalories (kcal)** | 13.45 (-399.5 , 429.3) | 35.31 (-398.5 , 443.6)^a,c^ | -97.19 (-533.4 , 287.4)^b^ | 40.64 (-343.4 , 482.7)^a^ | -10.77 (-386.6 , 378.9)^c^ | | **<0.001** | 71.02 (-401.1 - 490.8) | 96.82 (-375.3 , 488,5)^a^ | -312.24 (-608.8 , 171.8)^b^ | 207.41 (-347.9 , 586.1)^a^ | 14.24 (-550.8 , 485.8)^a,b^ | **0.001** |
| **ΔCarbohydrate (g)** | -10.51 (-67.8 , 43.6) | -0.98 (-60.7 , 55.6)^a^ | 34.05 (-8.4 , 87.3)^b^ | -54.73 (-109.8 , -5.3)^c^ | -11.01 (-51.7 , 28.8)^d^ | | **<0.001** | -8.01 (-75.3 - 48.8) | 4.09 (-68.5 , 63.9)^a,c^ | 24.95 (-14.4 , 109.8)^a^ | -47.4 (-108.4 , 19.9)^b^ | -26.46 (-62.2 , 20.4)^b,c^ | **<0.001** |
| **ΔProtein (g)** | 4.79 (-20.5 , 31.8) | 4.73 (-19.1 , 29.2)^a^ | -26.98 (-60.8 , -1.8)^b^ | 22.72 (-2.9 , 52.9)^c^ | -1.79 (-27.63 , 23.6)^d^ | | **<0.001** | 6.16 (-17.8 - 39.8) | 6.15 (-16.3 , 33.7)^a^ | -44.54 (-75.6 , -8.4)^b^ | 32.69 (1.3 , 59.5)^c^ | -0.91 (-33.4 , 38.8)^a^ | **<0.001** |
| **ΔLipid (g)** | -7.69 (-27.1 , 8.9) | -4.45 (-22.5 , 11.9)^a^ | -20.08 (-40.3 , -2.9)^b^ | -3.64 (-21.6 , 10.7)^a^ | -19.8 (-39.1 , -1.7)^b^ | | **<0.001** | -3.31 (-21.9 - 15.3) | -0.32 (-17.8 , 16.6)^a^ | -16.96 (-44.1 , 0.9)^b^ | -1.05 (-16.9 , 18.1)^a^ | -17.09 (-32.9 , -3.5)^b^ | **<0.001** |
| **ΔAlcohol (g)** | 0.01 (-1.1 , 1.7) | 0.01 (-0.8 , 1.1)^a^ | -0.01 (-2.1 , 1.4)^a^ | 0.02 (-0.6 , 2.7)^b^ | 0.02 (-1.7 , 4.2)^b^ | | **<0.001** | 0.01 (-1.1 - 1.0) | 0.01 (-0.8 , 0.9) | 0,01 (-1.6 , 0.1) | 0.01 (-1.0 , 1.1) | 0.01 (-1.8 , 2.2) | 0.581 |
| Data expressed as median and interquartile range (p25 , p75). Kruskal wallis test with post hoc Dunn-Bonferroni (Same letters do not differ from each other; different letters differ from each other). Values in bold are statistically significant (p<0.005). Δ = Data difference between follow-up and baseline. Glycated HB= Glycated hemoglobin. HC= Hip circumference. HDL=High-Density Lipoprotein. LDL = Low-Density Lipoprotein. METs = multiples of metabolic equivalents. TG= Triglycerides. WC = Waist circumference. | | | | | | | | | | | | | |
